# Emerging functions of the EGFR in cancer

**DOI:** 10.1002/1878-0261.12155

**Published:** 2017-11-27

**Authors:** Sara Sigismund, Daniele Avanzato, Letizia Lanzetti

**Affiliations:** ^1^ Fondazione Istituto FIRC di Oncologia Molecolare (IFOM) Milan Italy; ^2^ Department of Oncology University of Torino Medical School Italy; ^3^ Candiolo Cancer Institute FPO ‐ IRCCS Candiolo, Torino Italy

**Keywords:** cancer, EGFR, membrane trafficking, signal transduction

## Abstract

The physiological function of the epidermal growth factor receptor (EGFR) is to regulate epithelial tissue development and homeostasis. In pathological settings, mostly in lung and breast cancer and in glioblastoma, the EGFR is a driver of tumorigenesis. Inappropriate activation of the EGFR in cancer mainly results from amplification and point mutations at the genomic *locus*, but transcriptional upregulation or ligand overproduction due to autocrine/paracrine mechanisms has also been described. Moreover, the EGFR is increasingly recognized as a biomarker of resistance in tumors, as its amplification or secondary mutations have been found to arise under drug pressure. This evidence, in addition to the prominent function that this receptor plays in normal epithelia, has prompted intense investigations into the role of the EGFR both at physiological and at pathological level. Despite the large body of knowledge obtained over the last two decades, previously unrecognized (herein defined as ‘noncanonical’) functions of the EGFR are currently emerging. Here, we will initially review the canonical ligand‐induced EGFR signaling pathway, with particular emphasis to its regulation by endocytosis and subversion in human tumors. We will then focus on the most recent advances in uncovering noncanonical EGFR functions in stress‐induced trafficking, autophagy, and energy metabolism, with a perspective on future therapeutic applications.

**Abbreviations**
AGO2: Argonaute 2
AKT: AKT8 virus oncogene cellular homolog
AP2: adaptor protein 2
ATG14: autophagy‐related gene 14
Bcl‐2: B‐cell lymphoma gene 2
BRAF: v‐Raf murine sarcoma viral oncogene homolog B
Ca^2+^: calcium ion
Cbl: cellular homologue of Cas NS‐1 oncogene
CCP: clathrin‐coated pit
CLCb: clathrin light chain b
CME: clathrin‐mediated endocytosis
c‐MET: MET proto‐oncogene receptor tyrosine kinase
CRC: colorectal cancer
Dyn1: dynamin 1
EE: early endosome
EGF: epidermal growth factor
EGFR: epidermal growth factor receptor
ErbB: erythroblastosis oncogene B
ER: endoplasmic reticulum
ESCRT: endosomal sorting complex required for transport
FEME: endophilin‐mediated endocytosis
FGFR2: fibroblast growth factor receptor 2
GLUT1: Glucose Transporter Type 1
GLUT3: Glucose Transporter Type 3
GPCR: G‐protein‐coupled receptor
Grb2: growth factor receptor‐bound protein 2
HK1: hexokinase 1
ILV: intraluminal vesicle
KI: knock‐in
KO: knockout
LAPTM4B: lysosomal‐associated protein transmembrane 4 beta
LC3: microtubule‐associated proteins 1A/1B light chain 3B
LDLR: low‐density lipoprotein receptor
LIR: LC3‐interacting region
mAb: monoclonal antibody
MAPK: mitogen‐activated protein kinase
miRNA: microribonucleic acid
mTORC1: mechanistic target of rapamycin complex 1
mTORC2: mechanistic target of rapamycin complex 2
MVB: multivesicular body
MYC: myelocytomatosis oncogene cellular homolog
NCE: nonclathrin endocytosis
NSCLC: non‐small‐cell lung cancer
PDGFR: platelet‐derived growth factor receptor
PDK1: phosphoinositide‐dependent kinase‐1
PI3K: phosphoinositide 3 kinase
PIP_2_: phosphatidylinositol 4,5‐bisphosphate
PIPKIγi5: phosphatidylinositol‐4‐Phosphate 5‐kinase type Iγ
PKC: protein kinase C
PKCε: protein kinase C ε
PKM2: pyruvate kinase M2
PLC: phospholipase C
PM: plasma membrane
PTP1B: protein phosphotyrosyl phosphatase 1B
PUMA: p53‐upregulated modulator of apoptosis
Rab: Ras analog in the brain
Ras: retrovirus‐associated DNA sequence
RNF11: Ring Finger protein 11
RTK: receptor tyrosine kinase
RTN3: reticulon 3
SCD1: stearoyl‐CoA desaturase‐1
SGLT1: sodium‐glucose cotransporter 1
Src: Rous sarcoma oncogene cellular homolog
SREBP‐1: sterol regulatory element‐binding protein 1
SYNJ2: 5′‐inositol lipid phosphatase synaptojanin 2
TfR: transferrin receptor
TGF: transforming growth factor α
TNF‐α: tumor necrosis factor α
TPC: two‐pore channel
TXNIP: thioredoxin‐interacting protein
UV: ultraviolet
VPS34: vacuolar protein sorting 34

## 1 Introduction

The epidermal growth factor receptor (EGFR) belongs to the ErbB family of receptor tyrosine kinases (RTKs) and exerts critical functions in epithelial cell physiology (Schlessinger, 2014). It is frequently mutated and/or overexpressed in different types of human cancers and is the target of multiple cancer therapies currently adopted in the clinical practice (Yarden and Pines, 2012).

Early studies of the EGFR pathway started with the discovery of EGF in 1963 by Stanley Cohen and, later in the 1980s, of the EGFR gene. Since then, biochemical, structural, and genetic studies have depicted the molecular mechanisms underlying receptor transphosphorylation, which usually occurs in response to ligand stimulation, and the consequent activation of the intracellular signaling cascade. This cascade consists in the activation of multiple pathways that deliver the information from the cell surface, and the intracellular vesicular compartments, to the nucleus leading to the activation of genes responsible for cell proliferation, survival, and differentiation (Lemmon and Schlessinger, 2010; Schlessinger, 2014).

The best characterized functions of the EGFR are in the context of ligand‐ and kinase‐dependent activation, that is, the ‘canonical’ EGFR signaling pathway (Lemmon and Schlessinger, 2010). However, novel functions, both kinase dependent and independent, have been recently identified. They reveal unexpected roles of the EGFR, such as in the regulation of autophagy and metabolism (Tan *et al*., 2016a). These noncanonical functions are generally induced by cellular and environmental stresses. Several of these ‘stress pathways’ are activated in cancer cells to provide them with a survival advantage and resistance to therapy (Jutten *et al*., 2013; Tan *et al*., 2016a). This has led to an emerging concept that concomitant targeting of EGFR and stress pathways might offer a window of opportunity in cancer treatment.

## 2 Canonical ligand‐dependent EGFR signaling pathway

**Figure 1:**
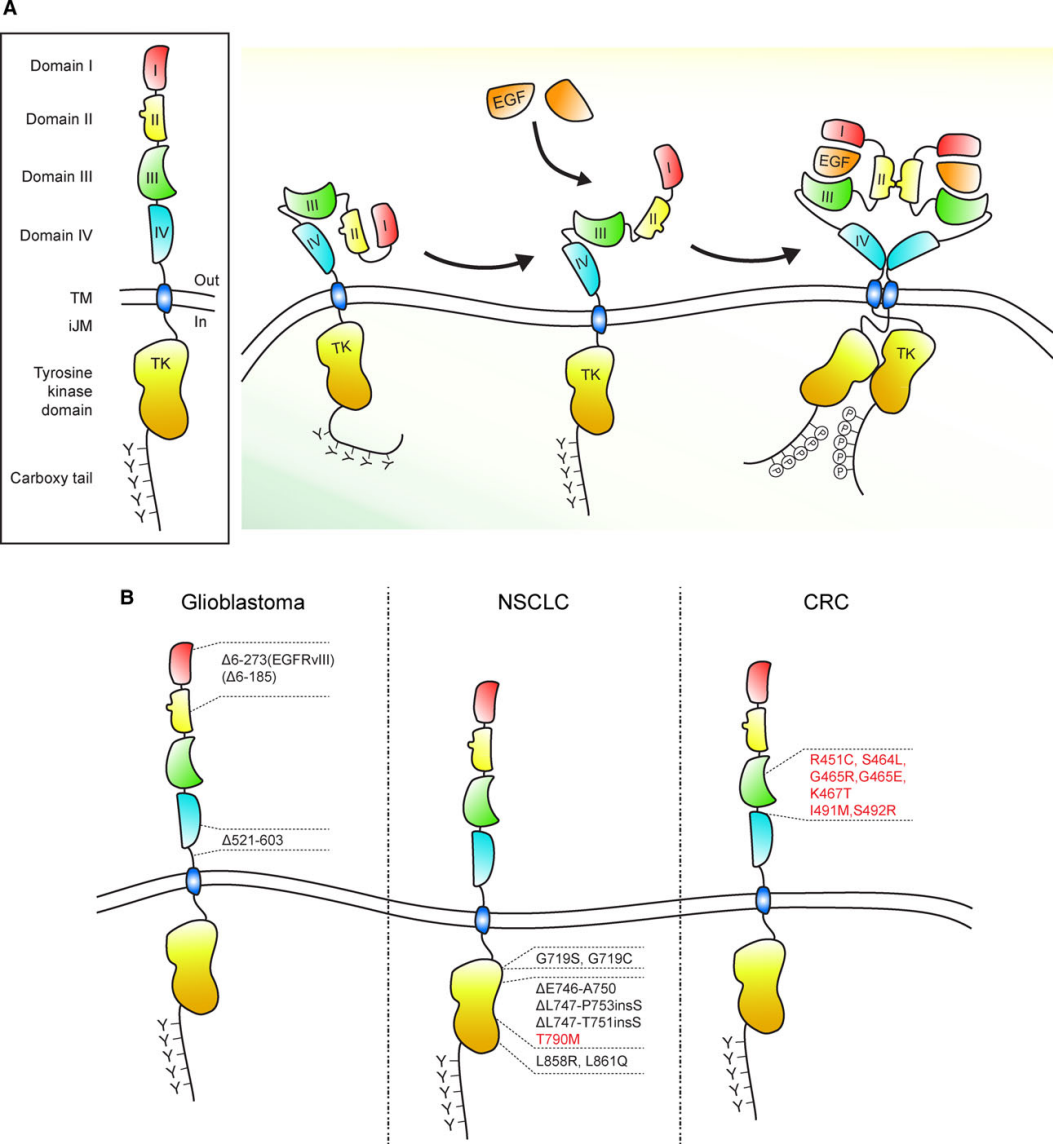
Scheme of EGFR and its mutations in glioblastoma and in lung and colorectal cancer. (A) Schematic representation of the EGFR and EGF‐induced receptor activation. The EGFR extracellular region encompasses domains I, II, III, and IV; following are the transmembrane region (TM), the intracellular juxtamembrane domain (iJM), the tyrosine kinase domain (TK), and the carboxyl‐terminal tail (carboxy tail). EGF binding to the receptor unmasks a dimerization motif and determines structural rearrangements that are conveyed to the cytoplasmic domain allowing the formation of asymmetric dimers between the two juxtaposed catalytic domains. (B) Most frequent EGFR mutations in glioblastoma, in NSCLC (non‐small‐cell lung cancer), and in CRC (colorectal cancer). Mutations found in tumors resistant to EGFR blockade are shown in red. In CRC, the indicated EGFR mutations have been identified in patients that progressed upon cetuximab treatment (Arena *et al*., 2015, 2016; Montagut *et al*., 2012; Van Emburgh *et al*., 2016).

Under unstimulated conditions, the EGFR is mainly found in an auto‐inhibited, dimerization‐incompetent, state at the plasma membrane (PM). Ligand binding promotes receptor dimerization, which determines a series of structural rearrangements that are conveyed to the cytoplasmic domain allowing the formation of asymmetric dimers between the two juxtaposed catalytic domains (Zhang *et al*., 2006; Fig. 1A). These events lead to the allosteric activation of the EGFR kinase and to the trans‐autophosphorylation of critical tyrosine residues in the cytoplasmic receptor tail, thereby triggering the signaling cascade (Lemmon *et al*., 2014). For in‐depth molecular details of EGFR activation, we refer the reader to recent reviews (Kovacs *et al*., 2015; Lemmon *et al*., 2014).

Ligand‐induced EGFR phosphorylation and conformational changes occurring in the intracellular tail lead also to the recruitment of the endocytic machinery that mediates receptor endocytosis, with internalization rates that are ~ 10‐fold higher for ligand‐bound than for unliganded EGFR (Conte and Sigismund, 2016).

The EGFR can heterodimerize with other ErbB family members, ErbB2, ErbB3, and ErbB4 (Lemmon *et al*., 2014), with critical effects on receptor trafficking and signaling (Lenferink *et al*., 1998). Indeed, heterodimers have a reduced ligand‐binding strength, leading to ligand dissociation in endosomes, and they are unable to recruit Cbl and the endocytic machinery as efficiently as EGFR homodimers (Baulida *et al*., 1996; Lenferink *et al*., 1998; Levkowitz *et al*., 1998; Waterman *et al*., 1999). Signaling from heterodimers is therefore enhanced and predicted to be more oncogenic than signaling from homodimers.

Seven EGFR ligands have been described to date, which have been shown to induce specific cellular responses and intracellular trafficking events both *in vitro* and *in vivo* (Roepstorff *et al*., 2009; Wilson *et al*., 2012; Yang *et al*., 2017). In some cases, these differences are determined by the different strength of the ligand–receptor interaction, which dictates whether the ligand dissociates (as in the case of TGFα transforming growth factor α) or not (as in the case of EGF) from the receptor in the mild acidic pH of the endosomes, favoring EGFR recycling or degradation, respectively. In other instances, the different signaling properties of the various ligands have been attributed to their ability to differentially stabilize the EGFR dimers, therefore determining specific signaling outputs (Freed *et al*., 2017).

Once activated at the PM, the EGFR also undergoes ubiquitination by the E3 ligase Cbl in complex with the adaptor molecule Grb2 (Levkowitz *et al*., 1998; Sigismund *et al*., 2013; Waterman *et al*., 2002). EGFR ubiquitination is threshold controlled by EGF concentration (Sigismund *et al*., 2013) and occurs on several lysine residues within the kinase domain (Huang *et al*., 2006). In specific cell contexts, EGFR ubiquitination works as a signal for receptor internalization into the nonclathrin endocytic (NCE) pathway. At later stages of trafficking, ubiquitination becomes a common requirement to target receptors to lysosomal degradation (see Section 2.1).

Ligand‐dependent EGFR activation transduces multiple signaling pathways, including the Ras/MAPK pathway, the PI3K/AKT pathway, and the phospholipase C (PLC)/protein kinase C (PKC) signaling cascade (Lemmon and Schlessinger, 2010). Canonical EGFR signaling is critical for several cellular functions including survival, proliferation, differentiation, and motility.

The quality, the amplitude, and the duration of these signaling events are tightly regulated by compartmentalization and trafficking of the EGFR along the endocytic pathway, as discussed in the following paragraphs.

### 2.1 Temporal regulation of EGFR signaling by endocytosis

The first step in the regulation of EGFR signaling takes place at the PM, where the EGFR is internalized through multiple endocytic pathways with different morphological, molecular, and kinetic features that influence receptor activity and fate (Barbieri *et al*., 2016; Bergeron *et al*., 2016). Both clathrin‐mediated endocytosis (CME) and several NCE pathways are involved in EGFR internalization (Barbieri *et al*., 2016). EGFR‐CME is active at all ligand concentrations in all type of cells (Carpentier *et al*., 1982; Goh *et al*., 2010; Hanover *et al*., 1984; Jiang *et al*., 2003; Sigismund *et al*., 2008; Sorkin and Carpenter, 1993). Conversely, the EGFR‐NCE pathways – despite their molecular and morphological differences – are generally activated at higher, but still physiologically relevant, EGF doses (≥ 10 ng·mL^−1^) and their significance is cell context dependent (Boucrot *et al*., 2015; Caldieri *et al*., 2017; Orth *et al*., 2006; also reviewed in Barbieri *et al*., 2016).

The molecular mechanisms underlying CME are well defined, with clathrin, adaptor protein 2 (AP2) and the large GTPase dynamin being the major players (see Kirchhausen *et al*., 2014; McMahon and Boucrot, 2011 for recent reviews). CME controls EGFR signaling through various mechanisms. At the PM, clustering of EGFR in clathrin‐coated pits (CCPs) is required to optimize receptor phosphorylation, and to amplify and spatially constrain EGFR signaling (Garay *et al*., 2015; Ibach *et al*., 2015). AP2 exerts a critical function during the assembly of CCPs and it is essential to maintain the right vesicle size, with predictable consequences for receptor clustering and signaling (Aguet *et al*., 2013; Kadlecova *et al*., 2017; Miller *et al*., 2015). In addition to AP2, dynamin and the cargo itself tightly regulate the timing of CCP assembly allowing for receptor clustering and productive signaling (Loerke *et al*., 2009). Some non‐small‐cell lung cancer (NSCLC) cells show an aberrantly accelerated CME, with deregulated CCP initiation and maturation (Chen *et al*., 2017). This phenotype has been linked to the activation of the neuronal dynamin isoform, dynamin1 (dyn1), in non‐neuronal cells, and/or to the overexpression of the clathrin light chain b (CLCb; Chen *et al*., 2017). The increased uncontrolled CME rate causes increased EGFR recycling and signaling through AKT, promoting cancer cell survival (Chen *et al*., 2017). Interestingly, both dyn1 and CLCb are upregulated in NSCLC and breast cancer (reviewed in Schmid, 2017).

In instances in which NCE is activated in parallel to CME, the integration of the two pathways is critical in determining the final signaling response. For instance, in HeLa and in other epithelial cells, CME and NCE determine opposing receptor fates (Sigismund *et al*., 2008): CME mainly induces receptor recycling (with limited EGFR degradation), while NCE – which requires EGFR ubiquitination as an internalization signal (Sigismund *et al*., 2005, 2013) – targets the majority of internalized EGFRs to degradation in the lysosome. In this way, CME, which is active at low EGF concentrations, directs the EGFR/EGF complex away from degradation and toward recycling to maintain signaling when ligand is limited. In addition, through recycling, CME also serves to prolong EGFR signaling, a requirement critical to achieve a productive proliferative response, and to polarize EGFR signaling to specific regions of the PM (Bisi *et al*., 2013; Sigismund *et al*., 2012). Polarized trafficking of cargo proteins to regions of the PM represents one of the most frequently altered functions of endo/exocytosis in cancer as it is primarily involved in migration and invasion of metastatic cells and in maintenance of epithelial cell polarity (reviewed in Lanzetti and Di Fiore, 2017).

EGFR‐NCE is activated only at high EGF concentrations and is critical for long‐term attenuation of EGFR signaling by directing EGFRs to lysosomal degradation. Recently, the mechanism governing EGFR‐NCE has been elucidated. This endocytic route depends on the function of an endoplasmic reticulum (ER)‐resident protein, reticulon 3 (RTN3), which is involved in the formation of contact sites between the ER and regions of the PM where EGFR‐NCE occurs (Caldieri *et al*., 2017, also discussed in Section 3.1). This modality of EGFR‐NCE appears to act as a safeguard against excessive EGFR signaling, and might represent a mechanism for modulating EGFR signaling at specific PM regions where polarized functions take place, an issue that deserves further investigation.

Other types of EGFR‐NCE occur at specific PM locations and are connected with cell migration. They include (a) the macropinocytic‐like pathway that originates, in mouse and human fibroblasts, from actin‐based membrane ruffles, defined as circular or dorsal ruffles (Orth *et al*., 2006), implicated in three‐dimensional cell motility and extracellular matrix degradation (Suetsugu *et al*., 2003), and (b) the fast endophilin‐mediated endocytosis (FEME). This latter pathway is involved in the internalization of several G‐protein‐coupled receptors and RTKs, including the EGFR, and is active at the leading edge of migrating cells, suggesting its involvement in polarized signaling during cell migration (Boucrot *et al*., 2015).

Once internalized, EGFRs reach the early endosomes (EEs), a further ‘level’ in the regulation of EGFR signaling. At this station, EGFRs are sorted toward different fates, recycling or degradation (reviewed in Wandinger‐Ness and Zerial, 2014). Receptor recycling is usually the default pathway. Escape from recycling is determined by EGFR ubiquitination, which is an active signal recognized by the ESCRT (endosomal sorting complexes required for transport) complexes that, through a stepwise process, sort receptors into multivesicular bodies (MVBs) and into lysosomes for degradation (reviewed in Raiborg and Stenmark, 2009; Wollert *et al*., 2009).

Besides sorting, endosomes work as platforms for EGFR signaling. Here, signals originating at the PM can be prolonged – in order to achieve a productive signaling response – and/or diversified – by assembling specific signaling complexes (reviewed in Villasenor *et al*., 2016). Furthermore, the endosome fusion and fission machinery tightly controls EGFR signaling by keeping the number of EGFR clusters per endosome constant over a wide range of EGF concentrations (Villasenor *et al*., 2015), thus conferring robustness to the system. Varying the number of EGFR clusters per endosome through alteration of the endosome fission/fusion rate critically impacts the EGFR signaling output, for example, proliferation vs. differentiation (Villasenor *et al*., 2015).

A novel regulatory mechanism occurring at the EEs has been recently described, which is able to sense the amount of EGFRs trafficking toward the endosomes and to induce *de novo* receptor biosynthesis and exocytosis, in order to preserve EGFR levels at the PM (Scharaw *et al*., 2016). When cells are continuously stimulated with high EGF doses, the transcription factor RNF11 translocates from the EEs to the nucleus where it induces transcription of genes required for EGFR transport to the PM (Scharaw *et al*., 2016). How RNF11 senses the amount of internalized EGFR at the EEs remains an open question.

### 2.2 EGFR cancer mutants divert from the normal trafficking itinerary

EGFR signaling is frequently altered in several human cancers due to *EGFR* gene amplification and/or protein overexpression, mutations or in‐frame deletions (Roskoski, 2014). The most frequent mutations in glioblastoma and lung cancer are illustrated in Fig. 1B; this figure also includes mutations found in colorectal cancers that are resistant to antibody‐mediated EGFR blockade]. These genetic lesions often occur concomitantly with increased EGFR ligand production due to autocrine or paracrine loops (Wilson *et al*., 2009, 2012). In many cases, EGFR genetic alterations determine abnormal EGFR trafficking, which contributes to increased signaling and tumor development. For instance, the increase in EGFR density at the PM due to EGFR amplification/overexpression was shown to stimulate receptor homo‐ and heterodimerization leading to kinase activation (Chung *et al*., 2010; Sawano *et al*., 2002; Wiley, 1988; Wilson *et al*., 2009). In particular, heterodimers with the ligand‐orphan receptor ErbB2 are constitutively active, evade receptor ubiquitination and degradation, and are mostly recycled back to the PM, thereby producing sustained signaling and cell proliferation (Mellman and Yarden, 2013; Schneider and Yarden, 2016). In agreement, saturation of the endocytic and/or the ubiquitination machinery has been proposed as a mechanism underlying sustained signaling in EGFR‐overexpressing cancer cells (Capuani *et al*., 2015; French *et al*., 1994; Wiley, 1988).

Oncogenic EGFR mutations and large genetic rearrangements (as observed in glioblastoma, brain, lung, breast, and ovarian cancers) often cause altered receptor endocytosis, which contributes to increased signaling properties (Yarden and Pines, 2012). In some cases, mutations directly disrupt the recruitment site of the E3 ligase, Cbl, in the intracellular domain of the receptor (i.e., EGFRvIV and EGFRvV mutants), thereby affecting receptor ubiquitination and lysosomal degradation (Roskoski, 2014). In other instances, mutations are located in the extracellular domain (i.e., EGFRvIII), leading to ligand‐independent receptor activation (Grandal *et al*., 2007; Han *et al*., 2006; Schmidt *et al*., 2003). Unexpectedly, these mutations also caused hypophosphorylation of the intracellular tyrosine residue 1045, the direct Cbl‐binding site, via an unknown mechanism. In this way, receptor ubiquitination and turnover are affected, resulting in sustained signaling (Grandal *et al*., 2007; Han *et al*., 2006; Schmidt *et al*., 2003). Somatic EGFR activating mutations have been detected in ~ 15–20% of NSCLC patients (Yun *et al*., 2007). One of the most frequent mutations, L858R, despite having a more highly phosphorylated Cbl‐binding site than the wild‐type receptor, is impaired in Cbl recruitment and receptor ubiquitination, again affecting trafficking toward the lysosome and receptor degradation, with consequent signal upregulation (Kon *et al*., 2014; Shtiegman *et al*., 2007). Increased heterodimerization of this mutant with ErbB2 has been proposed to cause this behavior (Kon *et al*., 2014).

Finally, it is important to stress that besides oncogenic alterations, inappropriate activation of the EGFR in cancer can originate from derailed receptor endocytosis and trafficking (Mellman and Yarden, 2013). This is achieved by two mechanisms: either mutated RTKs hijack the endocytic apparatus, which, in turn, fosters their signaling properties, or altered endocytic/trafficking genes potentiate the duration and the amplitude of the signal (Sigismund *et al*., 2012). Indeed, alterations in the balance between receptor recycling and degradation have been found in several aggressive cancers (Belle *et al*., 2015; Boulay *et al*., 2016). This latter mechanism largely relies on the overexpression and amplification of genes that are involved in RTKs endocytosis and recycling, including several GTPases belonging to the Rab family which control vesicular trafficking (Caswell *et al*., 2007; Cheng *et al*., 2004; Frittoli *et al*., 2014; Kajiho *et al*., 2016; Wheeler *et al*., 2015). Increased expression of endocytic/recycling molecules prolongs propagation of the signal and/or re‐locates RTKs and adhesive receptors at specific membrane sites, mainly involved in cancer cell invasion (Caswell *et al*., 2008; Eppinga *et al*., 2012; also reviewed in Lanzetti and Di Fiore, 2017; Mellman and Yarden, 2013; Mills *et al*., 2009; Mosesson *et al*., 2008; Sigismund *et al*., 2012). Among these molecules, copy number gain and overexpression of the 5′‐inositol lipid phosphatase synaptojanin 2 (*SYNJ2*) in breast cancer provides a paradigmatic example of sustained EGFR activation by altered trafficking pathways. Elevation of SYNJ2 promotes EGFR recycling at lamellipodia, stimulating cell motility and the formation of invadopodia (Ben‐Chetrit *et al*., 2015).

## 3 Noncanonical kinase‐dependent and kinase‐independent EGFR functions

In this section, we will discuss both kinase‐dependent and kinase‐independent functions of the EGFR that have recently emerged and that diverge from the canonical EGFR signaling pathway. For what concerns kinase‐independent roles, their existence has been known for many years. Indeed, while EGFR‐knockout mice are mid‐gestation or perinatal lethal (depending on the genetic background), due to gross developmental defects (Miettinen *et al*., 1995; Sibilia and Wagner, 1995; Threadgill *et al*., 1995), kinase‐dead EGFR‐knock‐in mice are viable, displaying only mild defects in the eye and skin (Luetteke *et al*., 1994). In addition, the EGFR is able to promote cell survival pathways through both kinase‐dependent and kinase‐independent mechanisms (Ewald *et al*., 2003; Tan *et al*., 2016a). These EGFR kinase‐independent functions could result from the heterodimerization of the EGFR with other ErbB family members or could be mediated by kinases that crosstalk with the EGFR pathway (e.g., Src or p38‐MAPK, see Section 3.2). Moreover, inactivation of phosphatases (e.g., PTP1B, see Sections 3.1 and 3.2) might contribute to activation of EGFR signaling. More work is needed to address whether these mechanisms are at play in living cells and whether they are mutually exclusive or coexisting in the regulation of EGFR function.

### 3.1 ER contact sites regulate EGFR signaling at different steps of the endocytic pathway

Communication between organelles is critical for several fundamental cellular processes, including organelle positioning and function, organelle fission, lipid transport, and Ca^2+^ signaling (van Bergeijk *et al*., 2016; Phillips and Voeltz, 2016; Saheki and De Camilli, 2017). Communication occurs through so‐called contact sites: regions of juxtaposition (≤ 20 nm) between two heterologous membranes, tethered by *in trans* protein–protein interactions (Eisenberg‐Bord *et al*., 2016; Phillips and Voeltz, 2016). In particular, the ER, due to its tubular organization that extends all over the cell, has been shown to make contact and to exchange materials with all of the other cellular organelles (Phillips and Voeltz, 2016).

**Figure 2:**
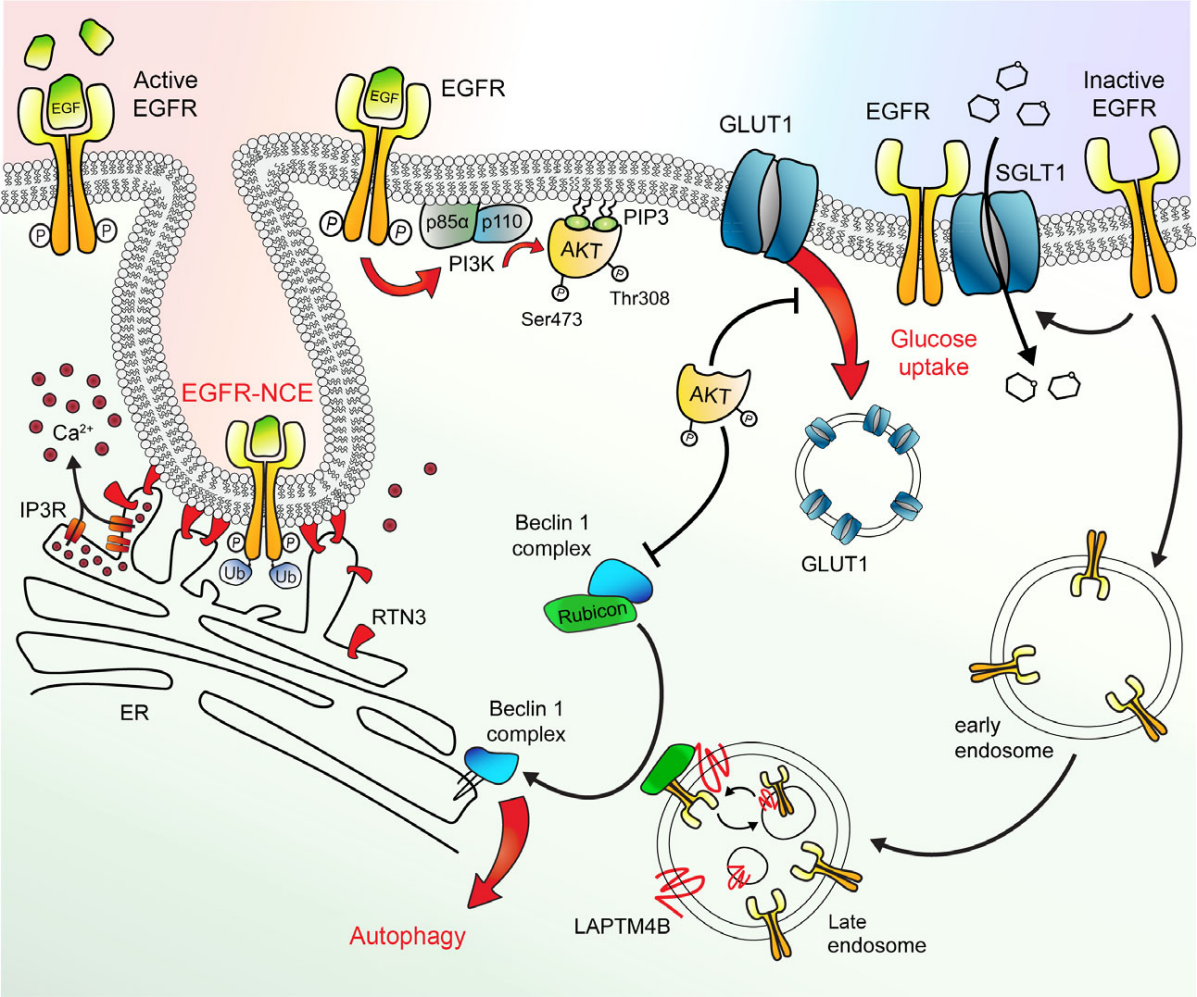
Active and inactive EGFR‐related functions. This picture schematizes some noncanonical EGFR functions. From left to right: EGFR stimulated with high EGF doses (active EGFR) is phosphorylated (P) and ubiquitinated (Ub) and undergoes both clathrin‐mediated endocytosis (not depicted) and nonclathrin‐dependent endocytosis (NCE), the latter dependent on the formation of RTN3‐mediated ER–PM contact sites. This is accompanied by calcium release in the proximity of contact sites, which likely controls fission of the tubular invagination. It is still unclear whether RTN3 is the tethering factor between the ER and the PM (as depicted), or it is just involved the tubulation of cortical ER, but not directly engaged at contact sites. EGFR ligand stimulation elicits the classical signaling cascade based on the recruitment of PI3K (made of its p85 regulatory subunit and p110 catalytic subunit) that catalyzes the formation of PIP3s. PIP3s bind to the PH domain of AKT and of phosphoinositide‐dependent kinase‐1, PDK1. PDK1 phosphorylates AKT on Thr308, while mammalian target of rapamycin complex 2, mTORC2 (not depicted here), is responsible for phosphorylation on Ser 473, leading to full AKT activation. Active AKT inhibits autophagy and blocks GLUT1 endocytosis. This latter function leads to higher levels of GLUT1 at the plasma membrane, increasing the uptake of glucose. In addition, ligand‐independent direct interaction of EGFR (inactive EGFR) and SGLT1 stabilizes the glucose transporter at the cell surface promoting high glucose uptake. Ligand‐unbound EGFR constitutively internalizes into early and late endosomes where it is sequestered by LAPTM4B. Here, the inactive EGFR interacts with Rubicon causing its dissociation from Beclin‐1. Beclin‐1 complex can now initiates autophagy on the ER membrane.

ER contact sites have a critical role in controlling EGFR signaling and trafficking at multiple steps. During the initial phase of endocytosis, high doses of EGF are able to induce tubulation of cortical ER and the formation of ER contact sites with the PM, at regions where the EGFR is internalizing via NCE (Caldieri *et al*., 2017; Fig. 2). The formation of these contact sites is critical to induce local Ca^2+^ signaling at ER–PM interface, which is in turn required for the fission of NCE tubular intermediates and, thus, for completion of the internalization process (Caldieri *et al*., 2017). This mechanism ultimately leads to EGFR endocytosis via NCE, receptor degradation, and signal termination (Caldieri *et al*., 2017; Sigismund *et al*., 2008). Polarized Ca^2+^ waves might also be critical in specifying the final EGFR‐NCE signaling output, given the role of Ca^2+^ in growth factor‐induced cell migration (Tsai *et al*., 2014), an issue that requires further investigation.

All along the endocytic route, the ER makes contact with the endosomes and these contact sites increase as endosomes traffic and mature (Friedman *et al*., 2013). ER–endosomal contact sites are critical in defining the timing and position of endosome fission during cargo sorting (Rowland *et al*., 2014), but they also have a direct role in the regulation of EGFR signaling. Indeed, a major RTK phosphatase, PTP1B, localizes to the cytosolic face of the ER and dephosphorylates the EGFR *in trans* during its trafficking to the endosomes/MVBs (Eden *et al*., 2010; Haj *et al*., 2002). Of note, PTP1B regulates constitutively internalized EGFR, thereby restricting spurious EGFR kinase activation, as well as ligand‐activated receptor that is dephosphorylated by PTP1B on the way to the lysosome (Baumdick *et al*., 2015). The formation of ER–MVB contact sites is mediated by annexin‐1 and is Ca^2+^ dependent (Eden *et al*., 2016; Kilpatrick *et al*., 2017). The release of Ca^2+^ occurs through the two‐pore channel that is localized on the endolysosomal membrane at ER contact sites (Kilpatrick *et al*., 2017). Disrupting these contact sites has been shown to delay PTP1B‐mediated EGFR dephosphorylation, causing delayed receptor degradation and enhanced signaling (Eden *et al*., 2016; Kilpatrick *et al*., 2017).

### 3.2 Stress‐induced EGFR trafficking pathways

Different stresses applied to cells have been shown to stimulate EGFR endocytosis and trafficking in a ligand‐independent fashion. For instance, UV radiation, cisplatin, inflammatory cytokines (tumor necrosis factor α), and the antibiotic anisomycin all trigger p38‐MAPK activation, required for ligand‐independent EGFR internalization (reviewed in Tan *et al*., 2016a; Tomas *et al*., 2014).

While the mechanism is similar for all these treatments, it has been most extensively characterized in the case of UV treatment. UV‐stimulated EGFR endocytosis occurs via CME and depends on the phosphorylation of serine/threonine residues in the C‐terminal receptor tail mediated by p38‐MAPK activity (Oksvold *et al*., 2004; Tomas *et al*., 2017; Tong *et al*., 2014; Vergarajauregui *et al*., 2006; Zwang and Yarden, 2006). Interestingly, other receptors, such as the insulin receptor, c‐MET, and the transferrin receptor, are not internalized upon UV treatment, suggesting the existence of some level of specificity (Zwang and Yarden, 2006). Once internalized, EGFRs accumulate in a subpopulation of MVBs, distinct from the EGF‐induced MVB pool, where they are entrapped into intraluminal vesicles (ILVs) without being degraded (Oksvold *et al*., 2002; Tomas *et al*., 2015). The process is reversible as, upon p38‐MAPK inhibition, ILV‐localized EGFRs can be recovered to the limiting MVB membrane from which they are recycled back to the PM (Tomas *et al*., 2015).

EGFR also responds to hypoxia, which, on the one hand, upregulates the transcription of the *EGFR* gene, providing a mechanism for EGFR overexpression in the absence of genetic alterations (Franovic *et al*., 2007); on the other hand, it triggers EGFR Src‐dependent, caveolae‐dependent endocytosis (Shen *et al*., 2013). At endosomes, EGFRs bind and phosphorylate the endosomal membrane‐associated protein, argonaute 2, a molecule involved in micro‐RNA (miRNA) maturation, causing inhibition of the maturation of tumor suppressor miRNAs, thus promoting cancer cell survival (Shen *et al*., 2013). A similar mechanism of EGFR internalization and endosomal accumulation appears to be at work also in the case of oxidative stress induced by H_2_O_2_ (Filosto *et al*., 2011; Khan *et al*., 2006; Ravid *et al*., 2002). In this case, the generation of reactive oxygen species inactivates redox‐sensitive, cysteine‐based, tyrosine phosphatases, including PTP1B, causing the activation of Src and, possibly, of the EGFR itself (Denu and Tanner, 1998; Lee *et al*., 1998). Src‐dependent caveolae‐mediated EGFR endocytosis is also activated by ionizing radiation. Importantly, mechanisms of resistance to ionizing radiation depend on the EGFR (Dittmann *et al*., 2008). Indeed, this treatment increases EGFR expression, induces Src activation and caveolae‐mediated EGFR endocytosis. Phosphorylation of threonine 654 in the EGFR juxtamembrane region by PKCε negatively regulates Cbl‐dependent ubiquitination and promotes EGFR nuclear translocation, leading to enhanced DNA repair and cell survival (Dittmann *et al*., 2008; Wanner *et al*., 2008). In agreement, EGFR nuclear localization has been associated with radiation resistance and poor clinical outcome (Tan *et al*., 2016a; Tomas *et al*., 2014).

In conclusion, it is emerging that multiple mechanisms of ligand‐independent trafficking are activated under stress conditions and that these mechanisms can promote cancer cell survival. However, more work is needed to molecularly dissect these pathways, in order to clarify how they are regulated, how they interplay with the canonical EGFR pathway, and whether they can be hijacked to prevent resistance to anti‐EGFR therapies.

### 3.3 Role of EGFR in autophagy

Autophagy is critical in maintaining cellular homeostasis and is finely regulated under physiological conditions to allow cells to rapidly respond to environmental changes. It is deregulated in different pathologies, including neurodegenerative diseases, aging, and cancer, and is one of the major mechanisms promoting resistance to cancer therapies (for recent reviews see, for instance Galluzzi *et al*., 2015, 2017; Goldsmith *et al*., 2014; Menzies *et al*., 2015; Rubinsztein *et al*., 2012).

The EGFR is a crucial regulator of autophagy. In nutrient‐rich growth conditions, ligand‐activated EGFR has a dual activity: on the one hand, it stimulates cell proliferation; on the other, it inhibits autophagy. Inhibition of autophagy is achieved: (a) directly, through the phosphorylation and consequent inhibition of Beclin‐1, a core subunit of the VPS34/autophagy initiation complex (Wei *et al*., 2013), and (b) indirectly, through the activation of AKT. In turn, AKT activates the mechanistic target of the rapamycin complex 1 (mTORC1) pathway, which ultimately inhibits autophagy (Tan *et al*., 2016a).

In contrast, under serum‐starved conditions, inactive EGFR is emerging as a promoter of autophagy. In this case, ligand‐unbound receptors, which constitutively traffic toward the endosomes, are sequestered by the lysosomal‐associated protein transmembrane 4 beta (LAPTM4B), localized in a subpopulation of early and late endosomes. The increased EGFR endosomal pool interacts with the autophagy inhibitor, Rubicon, causing its dissociation from Beclin‐1, leading to Beclin‐1 activation and autophagy initiation (Tan *et al*., 2015a,2015b; Fig. 2). This function is maintained by the kinase‐dead EGFR mutant, confirming that it is indeed independent of kinase activation (Tan *et al*., 2015b). The loss of EGFR generates cells defective in autophagy initiation, at variance with the loss of other RTKs, including c‐MET, PDGFR, and FGFR2 (Tan *et al*., 2015b), suggesting that this is an EGFR‐specific function.

Autophagy initiation seems to occur at ER–endosome contact sites. In particular, to initiate autophagy, autophagy‐related gene 14 on the ER surface has to interact with PIPKIγi5 kinase (PIPKIγi5K), an enzyme localized on endosomal membranes in complex with inactive EGFR and LAPTM4B. This binding stimulates phosphatidylinositol 4,5‐bisphosphate (PIP_2_) production by PIPKIγi5 and autophagy (Tan *et al*., 2016b). Thus, ER contact sites seem to provide a platform for autophagic complex assembly.

Interestingly, the ER‐resident protein RTN3, which is required for the establishment of ER–PM contact sites needed for EGFR endocytosis via NCE (Caldieri *et al*., 2017), has also been implicated in ER turnover by selective autophagy (Grumati *et al*., 2017). A specific RTN3 isoform, which possesses multiple LC3‐interacting regions, has been found to exert this function (Grumati *et al*., 2017). Whether these two functions of RTN3 are related, and how they are integrated within the cell, is not yet known; however, they might unveil connections between ligand‐dependent and ligand‐independent EGFR trafficking pathways.

Activation of autophagy has been found to promote resistance and survival of cancer cells treated with EGFR kinase inhibitors (Tan *et al*., 2016b). The mechanism seems to resemble the one induced by LAPTM4B in the physiological context. Indeed, these compounds promote endosomal accumulation of the EGFR, enhancing its association with Rubicon and favoring the dissociation of Rubicon/Beclin‐1 complex, thereby initiating the autophagic flux (Tan *et al*., 2015a). It is possible that other stresses causing EGFR endosomal accumulation (discussed in Section 3.2) might also activate autophagy as a part of their survival response, a scenario that deserves further investigation.

### 3.4 Mitochondrial functions of EGFR

The EGFR is usually considered to act at the PM and on vesicles mainly belonging to the endosomal compartment. However, it also localizes to the nucleus and mitochondria. Translocation of full‐length EGFR into the nucleus has long been documented and the functions it has at this location have been extensively investigated; we therefore refer the readers to detailed reviews (Brand *et al*., 2011; Han and Lo, 2012). Differently, the role of EGFR in mitochondria is more elusive and has been connected with antiapoptotic and metabolic functions.

In NSCLC cells, high levels of EGFR expression have been detected in the mitochondria (Che *et al*., 2015). In these cells, artificially mitochondria‐targeted EGFR redistributes these organelles to lamellipodia, increasing cell motility, possibly through the localized increase in energy (Che *et al*., 2015). In addition, translocation of wild‐type EGFR and of the EGFRvIII mutant into mitochondria has also been observed in cells treated with kinase inhibitors, or following proapoptotic stimuli (Cao *et al*., 2011). This translocation correlates with resistance to apoptosis and decreased sensitivity to EGFR inhibition (Cao *et al*., 2011). The latter function might be related to the ability of both wild‐type EGFR and EGFRvIII to constitutively bind to p53‐upregulated modulator of apoptosis (PUMA), a proapoptotic member of the Bcl‐2 family of proteins primarily located in the mitochondria (Zhu *et al*., 2010).

In breast cancer cells, translocation of EGFR to mitochondria has been shown to occur upon EGF stimulation resulting in phosphorylation of the cytochrome c oxidase subunit II (Boerner *et al*., 2004; Demory *et al*., 2009). The biological outcome of this modification is not clear. However, this event requires phosphorylation of the EGFR on tyrosine 845 by Src, which also undergoes mitochondrial translocation with similar kinetics to that of the EGFR (Demory *et al*., 2009). Of note, EGF stimulation also induces palmitoylation of mitochondrial EGFR, which, in turn, favors fusion of mitochondria (Bollu *et al*., 2014). EGFR, independently of its kinase activity, interacts with the fatty acid synthase, stimulating *de novo* synthesis of palmitate (Bollu *et al*., 2014). This finding points to the involvement of the EGFR in the regulation of cell metabolism and supports the existence of a signaling‐metabolic wiring that plays a critical role in cancer.

### 3.5 Role of EGFR in cancer cell metabolism

Oncogenic signaling pathways induce metabolic reprogramming in cancer cells supporting tumor growth (Cairns *et al*., 2011). In this context, EGFR signaling has been involved in the regulation of several metabolic processes that are critical for cancer cell proliferation: from the biosynthesis of fatty acids and pyrimidines, to glucose catabolism (Guo *et al*., 2009; Makinoshima *et al*., 2014). The EGFR promotes these metabolic pathways both directly by phosphorylating rate‐limiting enzymes (Lim *et al*., 2016; Zhang *et al*., 2017), or indirectly through activation of the MYC transcription factor and of the AKT signaling cascade (Babic *et al*., 2013; Guo *et al*., 2009; Makinoshima *et al*., 2014, 2015, and reviewed in DeBerardinis and Chandel, 2016; Masui *et al*., 2014).

In glioblastoma multiforme, oncogenic EGFR signaling by EGFRvIII stimulates the PI3K/AKT‐dependent nuclear translocation of sterol regulatory element‐binding protein 1 (SREBP‐1) and the expression of the low‐density lipoprotein receptor (LDLR). Increased LDLR, in turn, allows for the uptake of cholesterol bypassing negative feedback regulation (Guo *et al*., 2009). This represents a point of metabolic vulnerability as these cells depend on cholesterol uptake and are highly sensitive to inhibitors of fatty acid and cholesterol biosynthesis (Guo *et al*., 2011).

Furthermore, the EGFR has been recently found to directly phosphorylate and, thereby, stabilize stearoyl‐CoA desaturase‐1 (SCD1), resulting in the upregulation of monounsaturated fatty acid production (Zhang *et al*., 2017). Notably, phosphorylation of SDC1 correlates with poor prognosis of glioblastoma multiforme (Zhang *et al*., 2017), suggesting that it might have a causative role in these tumors.

One of the best‐studied metabolic drifts in cancer cells is the elevation of glycolysis in the presence of oxygen: the Warburg effect. Cancer cells are generally characterized by the avid uptake of glucose, which occurs through increased expression and membrane localization of glucose transporters, mainly GLUT1 and GLUT3 (Barron *et al*., 2016). Intracellular glucose is metabolized to pyruvate that, in cancer cells, is preferentially converted into lactate (Cairns *et al*., 2011).

The EGFR has been shown to foster aerobic glycolysis through several, both kinase‐dependent and kinase‐independent, mechanisms (Fig. 2). Physical association of EGFR with SGLT1 stabilizes the sodium‐glucose cotransporter at the cell surface increasing the glucose influx (Weihua *et al*., 2008). This kinase‐independent function provides survival advantages to cells, helping them escape autophagic cell death when grown in the presence of low glucose concentrations (Weihua *et al*., 2008).

In response to EGF stimulation, the EGFR controls expression of hexokinase (HK1) and phosphorylation of the pyruvate kinase M2 (PKM2), two glycolytic enzymes that catalyze key steps in the pathway, thus increasing aerobic glycolysis of breast cancer cells (Lim *et al*., 2016). One relevant ‘side effect’ of increased aerobic glycolysis is the production of high levels of lactate that, in these tumors, inhibits the cytotoxic activity of T cells, supporting their immune escape (Lim *et al*., 2016).

In lung adenocarcinoma cells bearing oncogenic EGFR mutations, deregulated signaling has been shown to stabilize GLUT1 at the cell surface through the activation of the PI3K/AKT/mTOR pathway (Makinoshima *et al*., 2015). Indeed, activation of AKT in response to cytokine stimulation has long been known to inhibit endocytosis of GLUT1 in lymphoid cells (Wieman *et al*., 2007; Wofford *et al*., 2008). Recent findings showing that AKT phosphorylates and inhibits thioredoxin‐interacting protein (TXNIP), the endocytic adaptor responsible for CME of GLUT1 (Hong *et al*., 2016; Waldhart *et al*., 2017), suggest that this might be the mechanism at work.

Of note, inhibition of the PI3K/AKT/mTOR pathway in lung cancer cells harboring EGFR mutations affects the glycolytic flux impairing their viability (Makinoshima *et al*., 2015). In line with these findings, combined inhibition of EGFR and glycolysis has been shown to synergistically suppress proliferation of triple‐negative breast cancer cells (Lim *et al*., 2016), further supporting the relevance of EGFR signaling in cancer cell metabolism.

### 3.6 Membrane trafficking influences the efficacy of EGFR‐targeted therapies

Given its critical role in cancer, several EGFR‐targeted therapies have been developed, including monoclonal humanized antibodies (mAbs) directed against the receptor extracellular domain, as well selective small‐molecule inhibitors targeting the tyrosine kinase domain. Small‐molecule EGFR inhibitors (e.g., gefitinib, erlotinib, and afatinib) have been approved for lung cancer treatment as a first‐line therapy in those cases where EGFR mutations have been confirmed (Cohen *et al*., 2005; Hirsch *et al*., 2013; Thatcher *et al*., 2005). Interestingly, in addition to kinase inhibition, gefitinib was shown to increase the formation of inactive EGFR dimers through some form of communication between the kinase domain and the extracellular dimerization domain, suggesting the possibility that gefitinib‐induced dimers could be more rapidly endocytosed and degraded (Arteaga *et al*., 1997; Gan *et al*., 2007), an issue that warrants further studies.

Cetuximab and panitumumab are the most widely employed EGFR‐neutralizing monoclonal antibodies, used for the treatment of head and neck cancer and metastatic colon cancer (Licitra *et al*., 2013; Peeters *et al*., 2015; Pierotti *et al*., 2010). Mechanistically, these compounds act by preventing ligand binding, thereby inhibiting receptor activation and downstream signaling (Bou‐Assaly and Mukherji, 2010; Dubois and Cohen, 2009; Vincenzi *et al*., 2008). They also favor EGFR dimerization, which, in turn, causes internalization of antibody‐bound dimers. These complexes are internalized at a lower rate and are more efficiently recycled to the PM compared with EGF‐bound dimers (Jaramillo *et al*., 2006). The combined use of anti‐EGFR antibodies directed against nonoverlapping antigens appears to be a more efficient strategy than the use of single antibodies, as it increases EGFR endocytosis and degradation (Ferraro *et al*., 2013; Friedman *et al*., 2005; Pedersen *et al*., 2010), raising the possibility of improving antitumor efficacy through the regulation of EGFR trafficking.

Currently, however, EGFR antibody‐based therapies, as well as small‐molecule inhibitors, have been shown to exert a limited response and to frequently evoke resistance in patients due to (a) secondary mutations within the EGFR itself (e.g., T790M in NSCLC, and mutations found in the extracellular domain of cetuximab‐resistant colorectal cancers, Fig. 1B), (b) alterations in other kinases (e.g., c‐MET, PIK3CA, BRAF, MAPK1), or (c) the emergence of feedback regulatory loops and mechanisms that overcome EGFR kinase inhibition (reviewed in Mancini and Yarden, 2016). In the latter case, the effect of therapies might be dampened by the activation of ligand‐independent EGFR trafficking pathways and functions, such as increased autophagy and elevated aerobic glycolysis (discussed in Sections 3.3 and 3.5). In addition, mechanisms that likely contribute to the emergence of drug resistance include also (a) relocalization of the EGFR to the nucleus following ionizing irradiation to promote DNA repair (Liccardi *et al*., 2011; Szumiel, 2006) and (b) translocation to mitochondria upon kinase inhibitor treatment to exert antiapoptotic effects (Cao *et al*., 2011; detailed in Section 3.4).

## Concluding remarks

The EGFR has long been considered the prototype of all RTKs. Indeed, most of the knowledge accumulated on signal transduction cascades in general and on the mechanisms underlying receptor endocytosis, recycling, and degradation has derived from studies focused on the EGFR. Nevertheless, novel unexpected functions of this receptor continue to emerge, some of which are linked to previously unrecognized subcellular localizations. Thus, despite the large body of knowledge already accumulated, this receptor still holds a number of surprises.

An emerging aspect that could be exploited for cancer treatment is the study of how membrane trafficking can influence the outcome of EGFR‐targeted therapies. Findings in this area could increase efficacy and overcome or delay the occurrence of resistance to treatments, an adverse event that invariably occurs in the patient population. Recently, in an attempt to overcome tumor resistance, simultaneous targeting of driver mutations and basic cellular processes has been proposed as a promising therapeutic perspective (Nagel *et al*., 2016). In this framework, endocytosis/recycling, autophagy, and metabolism might represent targets for the development of inhibitory tools to be tested in combination with EGFR inhibitors (Mellman and Yarden, 2013). A similar approach is currently being undertaken in tumors where the oncogenic EGFR signaling promotes metabolic reprogramming with promising results.
